# Preparation, characterization, and application of titanium nano-tube array in dye-sensitized solar cells

**DOI:** 10.1186/1556-276X-7-147

**Published:** 2012-02-21

**Authors:** Shih-Yu Ho, Chaochin Su, Chieh-Chung Cheng, Sasipriya Kathirvel, Chung-Yen Li, Wen-Ren Li

**Affiliations:** 1 Institute of Organic and Polymeric Materials, National Taipei University of Technology, Taipei, 10608, Taiwan, Republic of China; 2 Department of Chemistry, National Central University, Chung-Li, 32001, Taiwan, Republic of China

**Keywords:** TiO_2_, titanium tetrachloride, titanium (IV) *n*-butoxide, nano-tube, anodization, DSSCs

## Abstract

The vertically orientated TiO_2 _nanotube array (TNA) decorated with TiO_2 _nano-particles was successfully fabricated by electrochemically anodizing titanium (Ti) foils followed by Ti-precursor post-treatment and annealing process. The TNA morphology characterized by SEM and TEM was found to be filled with TiO_2 _nano-particles interior and exterior of the TiO_2 _nano-tubes after titanium (IV) *n*-butoxide (TnB) treatment, whereas TiO_2 _nano-particles were only found inside of TiO_2 _nano-tubes upon titanium tetrachloride (TiCl_4_) treatment. The efficiency in TNA-based DSSCs was improved by both TnB and TiCl_4 _treatment presumably due to the increase of dye adsorption.

## Introduction

Since O'Regan and Grätzel reported highly efficient TiO_2_-based dye-sensitized solar cells (DSSCs) in 1991, many attempts have been made to sensitize titanium dioxide (TiO_2_) nano-scale films. TiO_2 _nano-particulate films are typically preferred as they provide a high surface area for dye adsorption, leading to high photocurrent conversion efficiency. Due to the three-dimensional transport path, TiO_2 _nano-particulate films brought higher electron recombination and met larger grain boundary among interconnected nano-particles. In this research, we have fabricated vertically orientated one-dimensional nano-structure TiO_2 _nano-tube array (TNA) by electrochemical anodization. The TNA-based DSSCs were expected to have a better performance than the nano-particulate-based DSSCs due to the better electron transportation and recombination property. However, due to the less surface area of TiO_2 _nano-tube array, the efficiency of TNA-based DSSCs is still lower than that of TiO_2 _nano-particle-based DSSCs. Post-treatment of TNA by Ti precursors to form a TiO_2 _nano-particulate layers on TNA became a strategy which could increase the TiO_2 _surface area for more dye adsorption. This research showed that the DSSCs fabricated by TNA after post-treatment by titanium tetrachloride (TiCl_4_) and TiO_2 _nano-tubes after titanium (IV) *n*-butoxide (TnB) raised up the photocurrent conversion efficiency.

DSSCs have aroused intense interest over the past few years because they have been demonstrated to be able to achieve high solar-to-electric energy conversion efficiency with low-cost manufacture process and materials. In DSSCs, the photoelectrodes are made of porous semiconductor layers chemisorbed with an organic sensitizer. When DSSCs are illuminated with sun light, the photoelectron of the sensitizer is ejected into the semiconductor films and sent to the external circuit. The redox pairs in the electrolyte transport the holes from the oxidized dye molecules to the counter electrode to complete the electric cycle [1]. TiO_2 _is one of the most promising semiconductor materials in preparing the photoanodes for DSSCs due to its wide band gap characteristics and unique photoelectric properties [2]. TiO_2 _nano-particulate films are preferred as they provide a high surface area for dye adsorption, leading to high photocurrent conversion efficiency. The electron-collecting TiO_2 _layer in DSSCs is typically 10 to 15 μm thick with a three-dimensional network of interconnected nano-particles. However, TiO_2 _nano-crystalline films acquire long electron transport path and larger grain boundary between nano-particles [3,4]. This would hinder the electron collection efficiency and limit the performance of DSSCs. It was proposed that one-dimensional TNA aligned perpendicular to photoanode substrate could enhance the electron transportation and, thus, lower the possibility of electron recombination with redox electrolytes, leading to the higher photo-to-electron conversion efficiency [3-5]. The TNA has been first prepared by Zwilling et al. using the electrochemical anodization method [6]. The TNA morphology, including tube length, hole diameter, and wall thickness, can be systematically controlled by varying the anodization parameters, such as anodization potential, electrolyte, and pH value [7,8]. Zhu et al. had investigated the dynamics of electron transport and recombination properties of the oriented TiO_2 _nano-tube structure in DSSCs by frequency-resolved modulated photocurrent/photovoltage spectroscopies and found the higher charge-collection efficiency and slower electron recombination in the TiO_2 _nano-tube-based DSSCs than the TiO_2 _nano-particle-based counterparts [3]. One of the reasons for improving the performance of the DSSCs is considered to be due to the increase of the amount of the dye adsorbed onto the TiO_2 _surface of photoelectrodes in DSSCs. In order to increase the surface area of TiO_2 _electrodes, post-treatment of TNA to form an extra layer of TiO_2 _nano-particles has been applied [9-11]. In this work, we compared the effect of post-treatment of anodic TNA by different Ti-precursors on the TNA morphology and the resulting DSSCs performance.

## Experimental details

### Preparation, modification, and characterization of anodic TNA

Titanium foils with thickness of 0.25 mm (99.5% purity; Alfa Aesar, Ward Hill, MA, USA) were used for anodic growth of TNA. Titanium foils were first polished by sonication in chemical polishing solvent which contained nitric acid, ammonia fluoride, urea, ethanol, and hydrogen peroxide in 12:5:5:3:12 *v/v *ratio and rinsed subsequently with deionized (DI) water, acetone, and methanol. The anodization reaction was carried out in a two-electrode electrochemical cell with polished Ti foil (2 × 2.5 cm^2^) which served as the anode-working electrode and Pt foil (thickness 0.025 mm; Alfa Aesar) as the counter electrode. The separation between Ti electrode and Pt electrode was about 3.5 cm. The anodization electrolyte contains 0.3 wt% NH_4_F and 2 vol% H_2_O in ethylene glycol solution. The anodization was operated under a constant potential of 60 V at low temperature of 15°C with magnetic stirring. The reaction period controlled the thickness of TiO_2 _nano-tube arrays. Typically the TNA samples with tube length of approximately 15 μm were obtained after 2 h of anodization process. It is evident that increasing the TNA length leads to the increase of short-circuit photocurrent density due to the higher surface area available for dye adsorption. The TNA foils were then carefully washed with deionized water to remove the surface residual electrolyte in the nano-tube arrays. Such prepared TNA samples were then annealed at 450°C for 3 h with a heating rate of 1°C/min in order to transform the TNA from amorphous to anatase crystalline phase.

**Figure 1:**
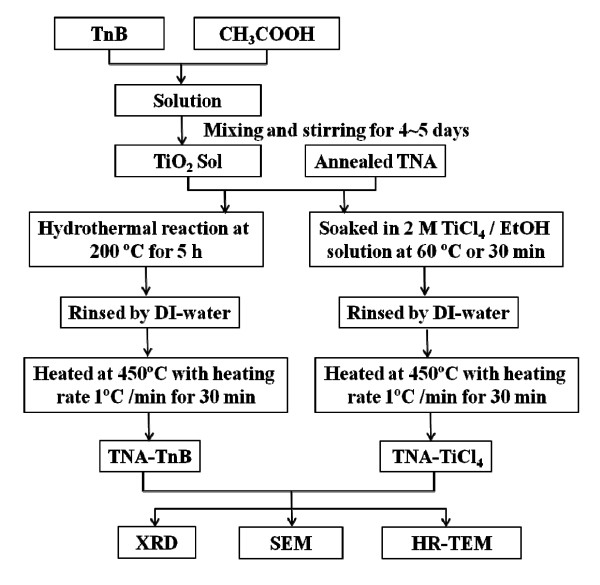
**Procedures for post-treatment of annealed TNA**. Fabrication flow chart of TNA-TiCl_4 _and TNA-TnB. TNA, TiO_2 _nano-tube array; DI-water, deionized water; XRD, X-ray diffraction; SEM, scanning electron microscopy.

Figure 1 summarizes the procedures for post-treatment of annealed TNA. The TiCl_4_-treated TNA (TNA-TiCl_4_) was prepared from annealed TNA which was soaked in 0.2 M TiCl_4 _solution (in ethanol) at 60°C for 30 min followed by heat treatment at 450°C for 30 min. The TnB-treated TNA (TNA-TnB) was prepared as follows: titanium (IV) *n*-butoxide (Ti(O-Bu)_4_, TnB) (ACROS Organics, New Jersey, USA) was mixed with 2 M CH_3_COOH (pH = 2.5) at room temperature under magnetic stirring for approximately 5 days until a homogeneous sol solution was obtained. The TiO_2 _sol and the annealed TNA were both transferred to a teflon-lined autoclave to perform the hydrothermal treatment at 200°C for 5 h. The Ti foil was then removed from the autoclave, rinsed with DI water, and heated at 450°C for 30 min to form TNA-TnB.

The surface morphology and crystal phase of TNA, TNA-TiCl_4_, and TNA-TnB were investigated by scanning electron microscopy (SEM) (SM6500F, JEOL Ltd., Akishima, Tokyo, Japan) and X-ray diffraction (XRD) (PANalytical X'Pert PRO, Almelo, The Netherlands), respectively. The results were confirmed by high-resolution transmission electron microscopy (Hitachi H-7100, Hitachi Ltd., Chiyoda, Tokyo, Japan).

### Dye-sensitized solar cell assemble and performance measurement

To fabricate DSSCs devices, three kinds of TNAs including TNA, TNA-TiCl_4_, and TNA-TnB, served as photoanodes, were combined with a transparent Pt counter electrode (cathode). The TNAs samples were sensitized by dye molecules (3 × 10^-4 ^M, N719 in a mixed solvent of acetonitrile and tertbutyl acohol (volume ratio = 1:1)) for 24 h. The amount of dye adsorbed on TNA electrodes was determined by desorbing the N719 from TNAs surfaces into a solution of 0.1 M NaOH. The concentration of the adsorbed N719 was analyzed by UV-visible spectrophotometer (V-630, JASCO Corp., Easton, MD, USA). The Pt cathode was made by a 'two-step dip coating' process developed by Wei et al. [12]. We have first prepared the poly-*N*-vinyl-2-pyrrolidone (PVP)-capped Pt nano-particles by dissolving PVP (M.W. = 8000) and H_2_PtCl_6 _(Pt precursor) into deionized water at room temperature and well stirred until a light-yellow solution was obtained. A NaBH_4 _solution was then added drop by drop to the H_2_PtCl_6_-PVP solution, and the solution quickly turned into a black color, indicating the formation of Pt nano-particles (Pt-PVP solution).

FTO glass (8Ω/sq., Solaronix SA, Aubonne VD, Switzerland) was pretreated by 1% ML-371 aqueous solution at room temperature for 1 min in order to increase adhesion between the PVP-capped Pt nano-particles and FTO surface. The ML-371-modified FTO substrate was then dipped into the Pt-PVP solution for 5 min and rinsed with deionized water followed by heat-treatment at 400°C for 5 h to remove completely the organic component and complete the preparation of counter electrode.

To assemble the DSSCs, the liquid electrolyte of 0.1 M lithium iodide, 0.05 M iodine (I_2_), 0.5 M 4-tert-butylpyridine, 0.5 M 1,2-Dimethyl-3-propylimidazolium iodide in acetonitrile was applied to the above-prepared Pt electrode which was then placed over the N719-coated TNAs electrodes. The edges of the cells sealed with a hot-melt film (Surlyn, 125 μm) and the electrolyte (I^-^/I_2_/I_3_^- ^redox couple) were injected into the space. The active cell area studied in this work is 0.25 cm^2 ^(0.5 cm × 0.5 cm). The photoelectrochemical performance of the resultant solar cells were measured by back illuminated through the Pt counter electrode due to the nonpenetration of light through the photoanode Ti metal substrate.

The current (I)-voltage (V) characteristics were performed using a digital source meter (Keithley model 2400, Keithley Instruments Inc., Cleveland, OH, USA) with the TNA-based DSSCs devices under one-sun AM 1.5 irradiation from a solar simulator (300 W Xe light and filters, Oriel Instruments, Irvine, CA, USA) on a 0.25 cm^2 ^sample area.

## Results and discussion

### Formation and characterization of anodic titanium oxide nano-tube arrays

**Figure 2:**
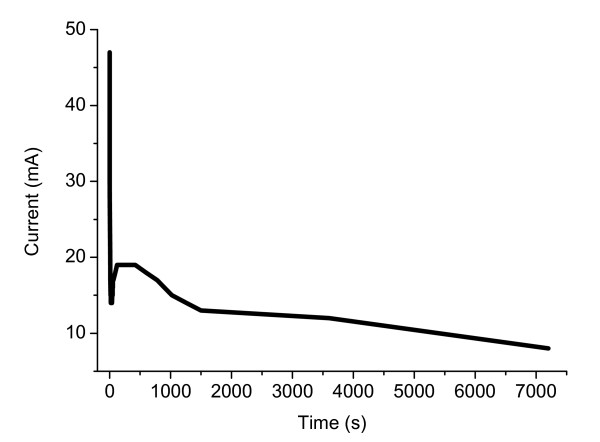
**Current-time plot of the constant potential anodization process**. Current-time plot recorded during the constant potential anodization process.

TiO_2 _electrode is one of the major concerns in DSSCs application. Since the TiO_2 _phase, morphology, and surface area of TiO_2 _films will affect the dye adsorption, electron transport, and electrolyte diffusion in the cell as well as the DSSCs performance. In this work, we have devised two kinds of Ti-precursor solutions to fabricate TiO_2 _nano-particles decorated TNA on Ti substrates which can serve as photoanodes for DSSCs device. During the optimization stage of anodization process, the important roles of the anodization condition, including the thickness of Ti-foil (0.05-0.25 mm), temperature (15-30°C), anodization potential (20-60 V), reaction period (10 min-24 h), and various kinds of F-containing electrolytes (HF, KF, and NH_4_F) in controlling the thickness (length of TiO_2 _nano-tubes), homogeneity, and morphology of TNA were revealed. The data presented below were obtained with NH_4_F/H_2_O/ethylene glycol electrolyte solution after anodization procedure at 15°C for 2 h. Figure 2 shows a typical current-time plot recorded during the constant potential anodization process. Within the first few seconds, the current dropped drastically to a local minimum indicating the oxidation of Ti-foil to form surface pits acting as nucleation sites for tube formation [13]. Upon increasing the pit density, the current increased to a maximum where the pit density reached saturation. After further anodization, the current gradually decreased due to the continuously lengthen of TiO_2 _nano-tubes. Such anodization behavior is commonly observed in the self-organized pore formation process [14] in which the competition between TiO_2 _oxide layer formation and dissolution of titanium progressed concurrently. Finally, the formation of vertically oriented TNA was achieved. The initially grown TNA was gray, which turned to yellow color after annealing at 450°C for 3 h.

**Figure 3:**
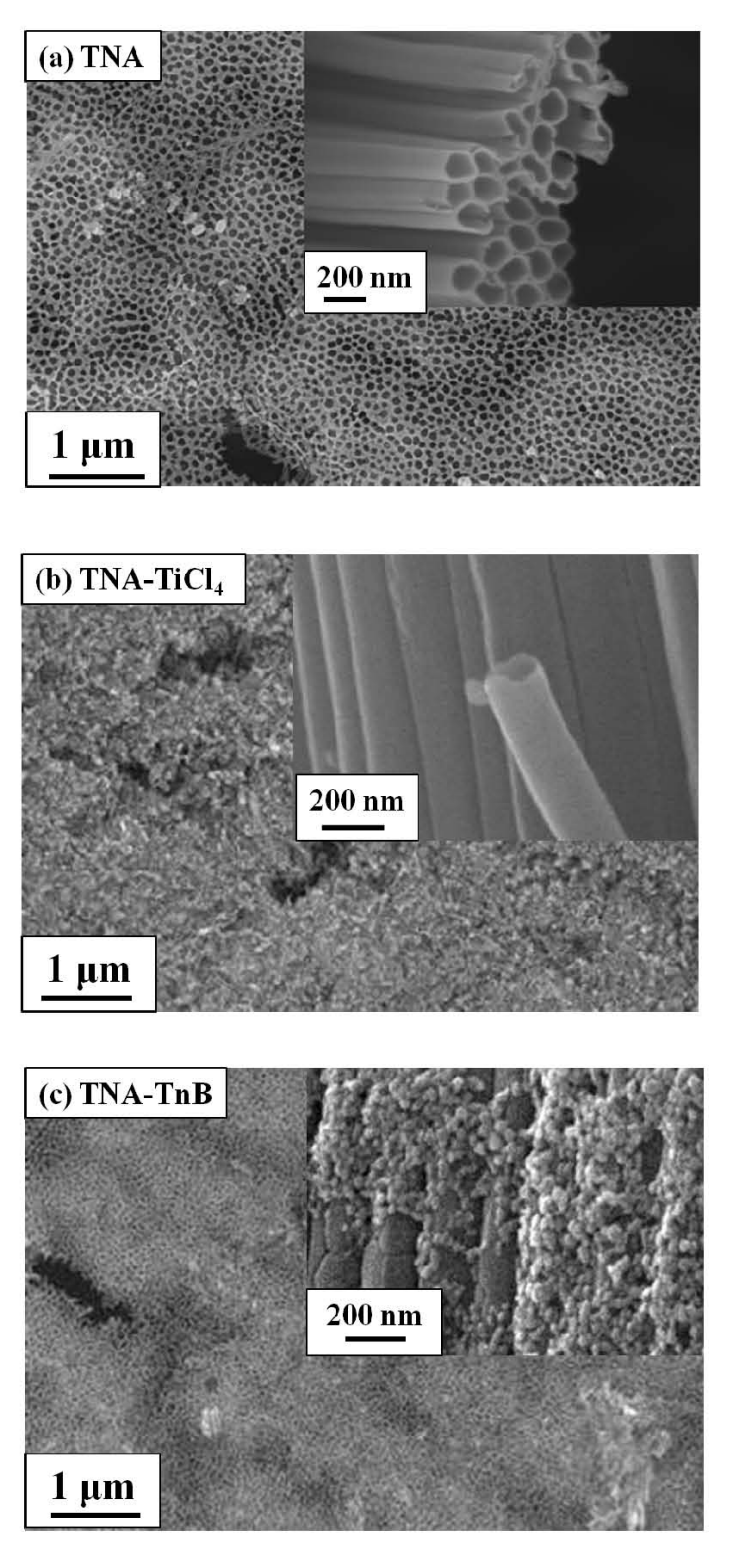
**SEM images of top view of (a) TNA, (b) TNA-TiCl_4_, and (c) TNA-TnB**. The inset images are the side view of (a) TNA, (b) TNA-TiCl_4_, and (c) TNA-TnB. TNA, TiO_2 _nano-tube array; TNA-TnB, TiO_2 _nano-tubes after titanium (IV) *n*-butoxide treatment; TNA-TiCl_4_, TiO_2 _nano-tubes after titanium tetrachloride treatment.

**Figure 4:**
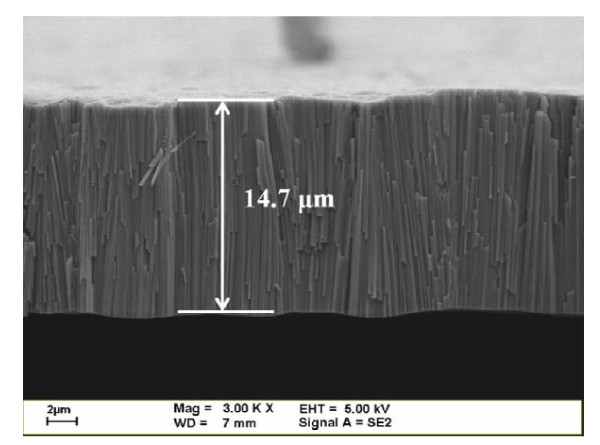
**SEM cross sectional view of annealed-TNA**. Mag, magnification; WD, width; EHT, extra high tension.

Figure 3a shows the SEM images of the untreated TNA formed by anodizing a titanium foil with two types of magnification: 10,000× (top view) and 50,000× (side view, inset). The SEM image of lower magnification of TNA (Figure 3a) shows high porosity character of anodic TiO_2 _films with some nano-scale cracks. The higher magnification image of TNA (inset of Figure 3a) shows the self-organized TiO_2 _nano-tubes aligned densely with hexagonal close-packed arrangement. The inner diameters of these TiO_2 _nano-tubes based on SEM images are in the range of 100-120 nm, and the wall thickness is approximately 10 nm. The thickness of TNA corresponding to the length of TiO_2 _nano-tubes is about 15 μm obtained from the SEM cross section analysis shown in Figure 4. It is consistent with other research works that high-aspect-ratio TiO_2 _nano-tubes can be fabricated with rapid growth rate by anodization [4,9,15].

**Figure 5:**
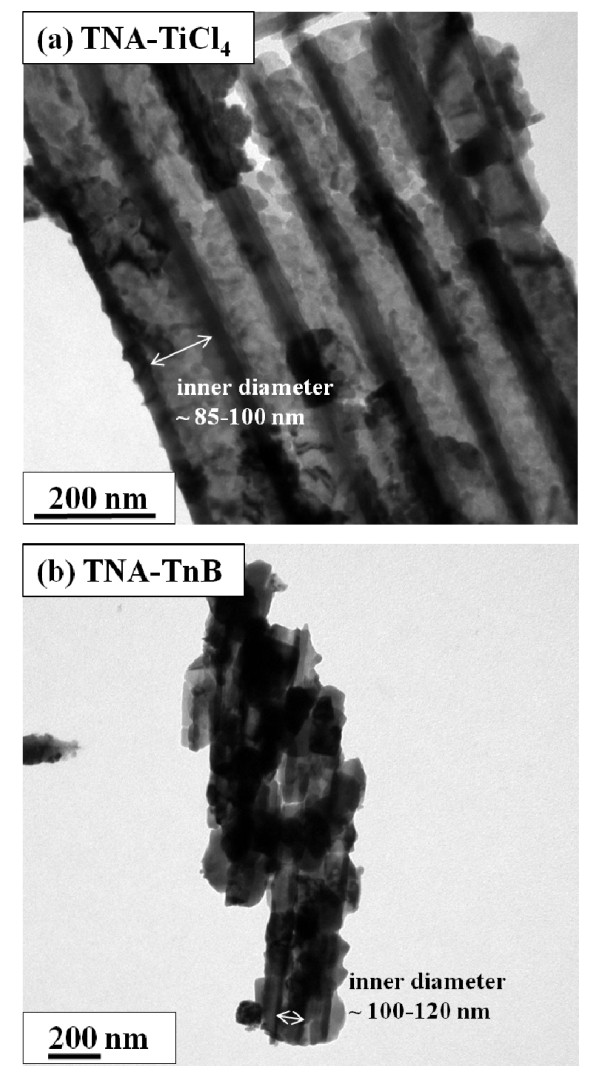
**TEM images of (a) TNA-TiCl_4 _and (b) TNA-TnB**. TEM, transmission electron microscope; TNA, TiO_2 _nano-tube array; TNA-TnB, TiO_2 _nano-tubes after titanium (IV) *n*-butoxide treatment; TNA-TiCl_4_, TiO_2 _nano-tubes after titanium tetrachloride treatment.

Figure 3b, c shows the top-view SEM images of the decorated TNAs after post-treatment by TiCl_4 _and TnB solution, respectively, with the corresponding side view images with higher magnification as shown in the inset of Figure 3b, c. The length of TiO_2 _nano-tubes remains the same after different post-treatment. For both TiCl_4 _and TnB treated TNAs samples (denoted as TNA-TiCl_4 _and TNA-TnB), additional materials can be observed on the top of TNAs and inside the TiO_2 _nano-tubes. Yet, outside the TnB-treated TNA, the TiO_2 _nano-tubes were apparently coated with an extra layer of TiO_2 _nano-particles. Transmission electron microscope (TEM) experiments had been performed on TNA-TiCl_4 _and TNA-TnB samples detached from the Ti foil and dispersed on a copper grid. The bulk crystallites were observed (Figure 5a) inside TiO_2 _nano-tubes in the case of TiCl_4_-treated TNA sample. In TnB-treated TNA, bulk crystallites were observed both inside and outside the nano-tubes as shown in Figure 5b. The average inner diameter of TiO_2 _nano-tubes in TNA-TiCl_4 _and TNA-TnB after different post-treatment was about 85-120 nm based on TEM analysis which is consistent with SEM results.

**Figure 6:**
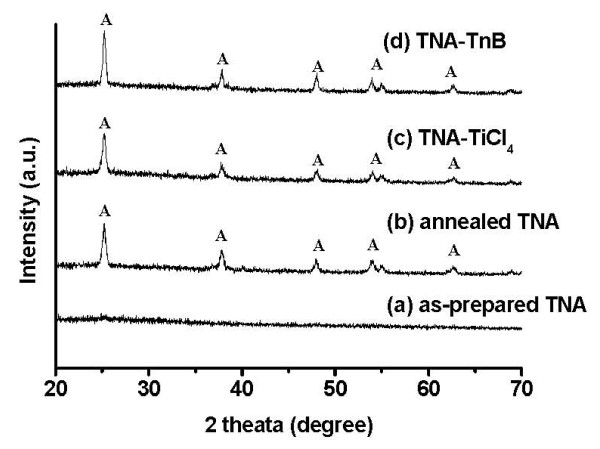
**XRD patterns of (a) as-prepared TNA, (b) annealed-TNA, (c) TiCl_4_-treated TNA, and (d) TnB-treated TNA**. XRD, X-ray diffraction; TNA, TiO_2 _nano-tube array; TNA-TnB, TiO_2 _nano-tubes after titanium (IV) *n*-butoxide treatment; TNA-TiCl_4_, TiO_2 _nano-tubes after titanium tetrachloride treatment; A, anatase diffraction peak.

XRD was used to confirm the crystalline phase of TiO_2 _nano-structure. Figure 6 shows the XRD patterns of (a) as-prepared TNA, (b) annealed-TNA, (c) TiCl_4_-treated TNA, and (d) TnB-treated TNA. The as-prepared TNA (before annealing) were amorphous (Figure 6a). Upon annealing to 450°C, the sharp anatase diffraction peaks appeared (Figure 6b) with crystal domain of approximately 20 nm. After post-treatment by TiCl_4 _and TnB, the XRD patterns remain the same for both TNA-TiCl_4 _and TNA-TnB, suggesting that the TiO_2 _crystalline phase was not affected by post-treatment. The slight increase of TiO_2 _crystal domain to 22 nm (for TNA-TiCl_4_) and 29 nm (for TNA-TnB) was due to the two times annealing at 450°C for post-treated TNAs samples.

### Application of anodic TNA electrodes to DSSCs and photoelectrochemical performance study

**Figure 7:**
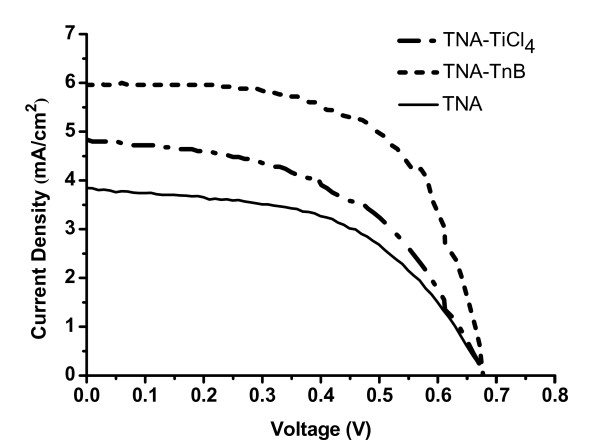
**Photocurrent-voltage characteristics of DSSCs made by TNA, TNA-TiCl_4_, and TNA-TnB**. TNA, TiO_2 _nano-tube array; TNA-TnB, TiO_2 _nano-tubes after titanium (IV) *n*-butoxide treatment; TNA-TiCl_4_, TiO_2 _nano-tubes after titanium tetrachloride treatment.

The above-prepared TiO_2 _nano-tube arrays (TNA, TNA-TiCl_4_, and TNA-TnB) were used to fabricate the DSSCs for photoelectrochemical performance study. The results obtained from I-V curve measurements for N719-sensitized DSSCs under simulated AM 1.5 illumination is shown in Figure 7. The thickness of TNAs layers on Ti foil was fixed at approximately 15 μm. Table 1 lists the TNAs thickness, the amount of dye adsorbed on TNAs layers (N719_ads_), and photoelectric data of the DSSCs in Figure 7 including the open circuit voltage, the short-circuit photocurrent density (J_sc_), the fill factor, and the photocurrent conversion efficiency (*η*). It is apparent that by post-treatment of the TNA, the performance of DSSCs was notably enhanced. The device based on the untreated TNA has shown the lowest J_sc _(3.84 mA/cm^2^) and *η *(1.38%). This is due to the low surface area of the untreated TNA which uptakes less amount of dye molecules (0.092 μmole/cm^2^). Higher efficiency and current density of DSSCs device might be attributed to the higher amount of adsorbed N719 and the fast electron transportation on TiO_2 _electrodes. As shown in Table 1, TnB treatment assisted the higher dye adsorption amount, raised from 0.092 to 0.116 μmole/cm^2 ^(approximately 26% increase of N719_ads_), and J_sc _also raised from 3.84 to 5.97 mA/cm^2 ^(approximately 55% increase of J_sc_). Both results lead to the efficiency improvement from 1.38% to 2.40% (approximately 74% increase of *η*). The significant increase of dye adsorption is due to the increased surface area from the decorated TiO_2 _nano-particles on TNA, as clearly seen from the SEM images (Figure 3c). TEM images (Figure 5b) also vindicated the presence of bulk TiO_2 _crystallites inside and outside the TNA. The further increase of J_sc _as well as *η *is attributed to the increase of TiO_2 _crystallinity of TNA-TnB, as evidenced by XRD. The dye loading for TNA-TiCl_4 _sample is lower compared to that of TNA-TnB because the TiO_2 _nano-particles only subsist inside the nano-tubes. The slightly higher efficiency of TNA-TiCl_4_-based DSSCs compared to the untreated TNA one is due to the better TiO_2 _crystallinity after two times annealing process. Variation in the performance of different devices might be due to the variations in the TiO_2 _tube length. Nevertheless, same trends were observed within each batch of device study.

**Table 1:** The I-V characterization of TNAs-based DSSCs

Sample	N719ads μmole/cm^2^	Film thickness (ηm)	Voc (V)	Jsc (mA/cm^2^)	FF	η (%)
TNA	0.092	14	0.68	3.84	0.53	1.38

TNA-TiCl4	0.096	15	0.66	4.83	0.51	1.61

TNA-TnB	0.116	16	0.66	5.97	0.61	2.40

## Conclusions

The decorated TNAs were successfully fabricated by anodization method followed by titanium precursor post treatment. The morphology of TNA without post-treatment was observed from SEM and TEM images, typically approximately 15 μm length, approximately 100 nm diameter, and 10 nm wall thickness were achieved after 2 h reaction. TNA with titanium precursor treatment alters the morphology which was confirmed from the SEM and TEM images. In the case of TNA-TnB, TiO_2 _nano-particles were filled interior and exterior of the TiO_2 _nano-tubes, whereas TiO_2 _nano-particles were filled only inside the TiO_2 _nano-tubes in TNA-TiCl_4 _upon TiCl_4 _treatment. An XRD pattern clearly indicates that the TNA, TNA-TiCl_4_, and TNA-TnB were pure anatase phase after annealing process at 450°C. The photocurrent conversion efficiency of TNA-based, TNA-TiCl_4_-based, and TNA-TnB-based DSSCs was 1.38%, 1.61%, and 2.40%, respectively. The results showed that the DSSC efficiency in TNAs was enhanced by TiCl_4 _and TnB precursor post-treatment, presumably due to the increase of dye adsorption. The higher solar efficiency in TnB-doped DSSCs is due to the formation of extra layer of TiO_2 _nano-particles on TNA, leading to the higher amount of dye adsorption as well as higher photocurrent.

## Abbreviations

DI: deionize; DSSCs: dye-sensitized solar cells; TEM: transmission electron microscope; TiCl_4_: titanium tetrachloride; TiO_2_: titanium dioxide; TNA: titanium dioxide nano-tube array; TnB: titanium (IV) n-butoxide; SEM: scanning electron microscopy; XRD: X-ray diffraction.

## Competing interests

The authors declare that they have no competing interests.

## Authors' contributions

The work presented here was performed in collaboration of all authors. SYH confirmed the results from the preliminary experiments and helped in writing the manuscript. CCC set up the anodization system and carried out the preliminary trails of anodization reaction. CS and WRL discussed the results and wrote the manuscript. SK and CYL proofread the manuscript and corrected the English. All authors read and approved the final manuscript.
